# A cross-validation of the provisional diagnostic instrument (PDI-4)

**DOI:** 10.1186/1471-2296-13-104

**Published:** 2012-10-15

**Authors:** Douglas E Faries, John P Houston, Ellen M Sulcs, Ralph W Swindle

**Affiliations:** 1 Lilly Research Laboratories, Indianapolis, IN, USA; 2 Department of Psychiatry, Indiana University School of Medicine, Indianapolis, IN, USA; 3 INC Research, Raleigh, NC, USA; 4 Harris Interactive, Rochester, NY, USA

**Keywords:** Cross validation, Diagnostic instrument, Anxiety, Depression, Hyperactivity, Mania

## Abstract

**Background:**

The Provisional Diagnostic Instrument (PDI-4) is a brief, adult self-report instrument for 4 common psychiatric diagnoses in primary care patients: major depressive episode (MDE), generalized anxiety disorder (GAD), attention deficit hyperactivity disorder (ADHD), and bipolar I disorder based on past or present mania. Our objective was to assess validity of the PDI-4 in a population independent of the study population originally used to develop the scale.

**Methods:**

An online version of the 17-item PDI-4 was administered to 1,047 adults in the US; respondents also completed the PHQ-9, HADS-A, CAARS-S, and MDQ within the online survey. Respondents self-reported diagnosis by a healthcare professional with the terms depression (n=221), anxiety (n=218), attention deficit disorder (n=206), bipolar or manic depressive disorder (n=195), or none of these (n=207). Statistical analyses examined convergent and discriminant validity, and operating characteristics of the PDI-4 relative to the individual, validated, self-rated scales PHQ-9, HADS-A, CAARS-S, and MDQ, for each PDI-4 diagnosis.

**Results:**

Convergent validity of the PDI-4 was supported by strong correlations with the corresponding individual scales (range of 0.63 [PDI-4 and MDQ] to 0.87 [PDI-4 and PHQ-9]). Operating characteristics of the PDI-4 were similar to results in the previous site-based study. The scale exhibited moderate sensitivities (0.52 [mania] to 0.70 [ADHD]) and strong specificities (0.86 [mania] to 0.92 [GAD]) using the individual scales as the gold standards. ANOVAs demonstrated that PDI-4 discriminated between subsets of patients defined by pre-specified severity level cutoff scores of the individual scales. However, overlapping symptoms and co-morbidities made differentiation between mental diagnoses much weaker than differentiation from the control group with none of the diagnoses.

**Conclusions:**

The PDI-4 appears to be a suitable, brief, self-rated tool for provisional diagnoses of common mental disorders. However, the high level of symptom overlap between these diagnoses emphasizes that such brief scales are not a replacement for thorough diagnostic evaluation by trained medical providers.

## Background

The need for accurate diagnoses is great due to the rate of mental disorders in the general population and the consequences of undiagnosed or incorrect diagnoses. Previous estimates have suggested 20% of primary care patients have a mental disorder, and these often go undetected [1] resulting in reductions in quality of life, increased healthcare costs, decreased productivity, and greater functional impairment [2-6]. The prevalence rates of the PDI-4 diagnoses in primary care populations are quite high: 8% to 15% for adult attention deficit hyperactivity disorder (ADHD) [7,8], 10% for bipolar spectrum disorders [9], 4% to 19% for major depressive episode (MDE) [10,11], and 7% to 15% for generalized anxiety disorder (GAD) [10,12].

The PDI-4 ( Additional file 1)is a patient-completed, diagnostic tool developed to assist in the determination of the following 4 psychiatric diagnoses often encountered in primary care settings: MDE, GAD, ADHD, and bipolar I disorder based on a history of past or present mania [13]. The scale consists of 17 items: 4 assessing frequency of specific symptoms from each of the 4 targeted diagnoses, and a final question assessing the impact on functioning. For consideration of each diagnosis, the patient must have at least 3 symptoms at or above the specified cutoff frequency [13]. The tool was created to assist with the diagnoses of mental disorders in primary care settings, where time constraints typically limit the ability to utilize healthcare professional-administered diagnostic interviews. Screening for mental disorders is often complicated by overlapping symptoms, co-morbid diagnoses, and need for extensive training with diagnostic instruments. While several validated patient-rated scales exist that are used as screening instruments for specific diagnoses, a single tool is needed to screen for multiple, common diagnoses due to co-morbidities and overlapping symptoms.

The initial validation of the PDI-4 was conducted in an in-person study of 704 patients conducted within primary care office settings [14]. Based on this study, the scoring rules for the scale were created, and operating characteristics of the scale were estimated in relation to gold standard diagnostic interviews (Structured Clinical Interview Research Version [SCID] for *Diagnostic and Statistical Manual of Mental Disorders, Fourth Edition* [DSM-IV] axis I disorders and the Adult ADHD Clinician Diagnostic Scale version 1.2 [ACDS] [15,16]). Houston et al. observed respective sensitivities and specificities of 83% and 75% for GAD, 80% and 80% for MDE, 83% and 82% for mania, and 82% and 73% for ADHD [14]. Concurrent validity was established by demonstrating expected correlations with existing validated scales for the specific diagnoses, and the impact of elevated scores on the PDI-4 was demonstrated by impairment measured by the Mental Component Score and the Physical Component Score on the 12-item Medical Outcomes Study Short-Form Health [17]. Houston et al. [14] later studied the properties of the PDI-4 for additional anxiety-related diagnoses. However, cross-validation of the scale was needed due to the small sample sizes for select populations. The scoring rules and operating characteristics were based on the same primary care-based sample; therefore, the instrument and its operating characteristics needed to be examined in an independent sample and in a more diverse patient population.

In the current study, a cross-sectional survey was administered to provide additional data to assess the validation of the PDI-4 and its generalizability to a more diverse population in a non site-based setting.

## Methods

### Study design and participants

This study was a cross-sectional survey of 5 cohorts of adults in the United States (US). Four of the cohorts were patients selected due to self-report of a previous diagnosis by a healthcare professional with at least 1 of the 4 disorders corresponding to the PDI-4: depression for MDE, anxiety for GAD, attention deficit disorder for ADHD, and bipolar or manic depressive disorder for past/present mania. The fifth cohort was a random sample of patients that did not self-report any of the 4 diagnoses. Harris Interactive participated in the design and administration of the survey in their pre-established national participant panel.

Participants were at least 18 years old, had at least a sixth grade education, and were recruited from the closed-membership Harris Chronic Illness Panel (a subset of the multi-million member Harris Pole Online Panel). Participants were sampled so that they represented all major US regions, with a goal of including at least 20% elderly patients, no more than 65% men or women, and an adequate minority representation. Potential participants received an e-mail invitation to a single-use, password-protected survey site. The overall goal was to obtain survey data from 1000 participants with approximately 200 from each cohort. This sample size was selected because it would provide sufficient power and sensitivity for each of the planned analyses (ie, provide planned accuracy for confidence intervals for concurrent validity and provide power for effect-size differences in known groups validity). The survey screened respondents for inclusion in the study, and those who qualified gave consent via a checkmark within the survey prior to their inclusion in the study. The Copernicus Group Institutional Review Board approved the conduct of the study.

### Questionnaire

The survey administered to all patients consisted of 78 items and was estimated to take approximately 15 minutes to complete. Items included the following: demographics and other participant characteristics (age, gender, race, education, income, mental disorder diagnoses, mental disorder medication use, marital status, employment status, geographic region, and recent physician visits); the 17 items from the PDI-4 scale, and items from comparator-validated, patient-rated scales for each of the following diagnoses: the Patient Health Questionnaire (PHQ-9) for MDE, the Anxiety subscale of the Hospital Anxiety and Depression Scale (HADS-A) for GAD, the Conners’ Adult ADHD Rating Scale Self Report (CAARS-S) for ADHD, and the Mood Disorder Questionnaire (MDQ) for mania. These comparator scales were selected because they are well-studied, validated, self-report scales that allow comparisons with previous work; these scales were all used in the initial study of the PDI-4 [1,14]. The PHQ-9 is a 9-item, participant-reported scale that parallels the 9 DSM-IV diagnostic symptoms of depression [18]. The HADS was originally developed to identify depression and anxiety symptoms in a non-psychiatric hospital setting and includes two 7-item subscales, with only the anxiety subscale (HADS-A) used in this study [19]. The CAARS-S Screening Version assesses symptoms and behaviors related to adult ADHD [20]. The DSM-IV Symptoms subscale of the CAARS-S was used in this study. The MDQ is a 15-item scale assessing past and present mania [21].

### Statistical methods

Descriptive statistics were used to summarize the population of subjects completing the survey as well as the overlap in symptoms across the 4 diagnoses. The analysis of the PDI-4 included assessment of concurrent validity and known groups validity (discriminant), and a summary of the operating characteristics of the tool in this population.

Each symptom item of the PDI-4 was assigned a score of 0, 1, 2, 3, or 4, depending on symptom frequency endorsed. Concurrent validity was assessed using correlations among the resulting 4-item score sum for each of the four PDI-4 subscales and the validated scale for each diagnosis (PHQ-9, MDQ, CAARS-S, and HADS-A). It was hypothesized that the PDI-4 subscales would correlate strongly with the corresponding validated scale (eg, PDI-4 MDE with the PHQ-9) and positively but less strongly with the other subscales and scales, providing similar results to those seen in the initial validation study.

The sensitivity, specificity, positive predicted value, and negative predicted value of each PDI-4 subscale were computed. As formal diagnostic interviews were not performed in this study, the corresponding patient scales with published cutoff scores (PHQ-9 score ≥ 12, HADS-A score ≥ 14, CAARS-S score ≥ 28, and MDQ score ≥ 7 with functioning item score of ≥ 3) were used to establish a “diagnosis” for PDI-4 comparison for the calculations of sensitivity, specificity, and positive and negative predicted value calculations. These cutoff scores were previously used by Houston et al. [13] in primary care patients and produced the following operating characteristics for each scale (sensitivity, specificity) relative to structured diagnostic interviews: PHQ-9 for MDE (0.79, 0.81), HADS-A for GAD (0.54, 0.81), CAARS-S for ADHD (0.54, 0.87), and MDQ for mania (0.83, 0.63).

Known groups (discriminant) validity was summarized using analysis of variance (ANOVA) models for each of the four PDI-4 subscales. Each model utilized a PDI-4 subscale as the outcome measure, with terms for cohort (based on the corresponding diagnosis), gender, age, and race. It was hypothesized that groups with the diagnosis would have significantly higher PDI-4 scores than patients without the corresponding diagnosis, where the diagnosis cohorts were determined by an independent measure. Specifically, cohorts were diagnosed based on published cutoff scores for the PHQ-9 (score ≥ 12), HADS-A (score ≥ 14), CAARS-S (score ≥ 28), and MDQ (score ≥ 7 with functioning item score of ≥ 3). For the assessment of the PDI-4 depression subscale, the ANOVA model was utilized to detect differences using the PDI-4 subscales between cohorts with PHQ-9 depression versus subjects without PHQ-9 depression. The negative group was then subdivided into participants with none of the other 3 diagnoses and participants with at least 1 of the other 3 diagnoses. Similar models were conducted for the PDI-4 GAD, ADHD, and mania subscale scores.

## Results

**Figure 1:**
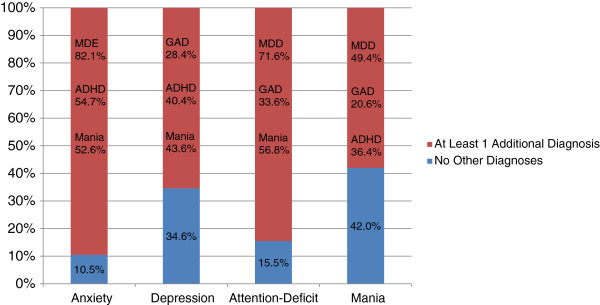
**Summary of overlapping symptoms based on PHQ-9, HADS-A, CAARS-S, and MDQ screening cutoff points.** Abbreviations: ADHD = attention deficit hyperactivity disorder; Anx = anxiety; GAD = generalized anxiety disorder; MDD = major depressive disorder. The labels on the x-axis denote patients meeting the cutoff score for the corresponding patient-rated scale: PHQ-9 score ≥ 12 for MDE, HADS-A score ≥ 14 for GAD, CAARS-S score ≥ 28 for ADHD, MDQ score ≥ 7 with functioning item score of ≥ 3 for mania. The same definitions are used in computing the percentages of patients with other diagnoses denoted inside each histogram bar.

The sample size targets were met with 1,047 subjects completing the online interview in the second half of 2010, and approximately 200 of them were within each cohort. Table 1 summarizes the characteristics of the sample population, including scores on the PDI-4 and corresponding scale. Cohorts for this initial summary were based on the patients’ self-report of diagnoses given to them by their physician. The overall sample was approximately 60% female and 84% Caucasian, with an average age of 51 years. Figure 1 describes the co-morbidities and overlapping symptoms in the sample by defining GAD, MDE, ADHD, and mania using published cutoff scores from the validated scales (PHQ-9, HADS-A, CAARS-S, and MDQ). The high prevalence of overlapping symptoms is apparent. For instance, among patients meeting the diagnosis cutoff score for GAD based on the HADS-A, 89.5% also equaled or exceeded the diagnostic cutoffs for 1 or more additional conditions (MDE by PHQ-9, ADHD by the CAARS-S, and/or mania by the MDQ). Indeed, 82% met or exceeded the PHQ-9 cutpoint for MDE.

**Table 1:** Summary of demographics and clinical characteristics of the population: by self-reported diagnosis cohort

**Variable**	**Total Group (N=1,047)**	**Anxiety (N=218)**	**Depression (N=221)**	**ADHD (N=206)**	**Manic Depressive (N=195)**	**Control (N=207)**
Gender (% female)	59.6%	59.6%	59.3%	51.0%	66.2%	62.3%
Age, years (SD)	51.4 (16.5)	50.3 (17.7)	51.4 (17.1)	51.5 (14.4)	52.1 (12.1)	51.5 (19.7)
Ethnicity (% Caucasian)	84.1%	88.5%	81.5%	87.3%	89.2%	73.0%
Education (% completed high school)	98.5%	97.6%	99.5%	98.5%	98.5%	98.5%
Income (mode)	$35,000 to $49,000	$35,000 to $49,000	$35,000 to $49,000	$50,000 to $74,000	$35,000 to $49,000	$35,000 to $49,000
PDI-4 GAD, mean (SD)	7.0 (3.6)	8.0 (3.5)	7.2 (3.0)	7.3 (3.4)	8.5 (3.3)	4.2 (3.2)
PDI-4 MDE, mean (SD)	5.9 (3.9)	6.7 (3.8)	6.5 (3.3)	5.8 (3.7)	7.9 (3.9)	2.9 (2.9)
PDI-4 ADHD, mean (SD)	4.9 (3.1)	4.9 (2.9)	4.8 (2.7)	5.8 (2.9)	6.4 (3.2)	2.8 (2.5)
PDI-4 Mania, mean (SD)	5.2 (3.7)	4.7 (3.1)	4.3 (3.1)	6.4 (3.5)	7.9 (3.8)	3.1 (2.9)

Abbreviations: *ADHD* = attention deficit hyperactivity disorder; *GAD* = generalized anxiety disorder; *MDE* = major depressive episode; *PDI-4* = Provisional Diagnostic Instrument; *SD* = standard deviation.

Table 2 summarizes concurrent validity by providing the correlations of the PDI-4 subscales with the corresponding diagnosis rating scales. As hypothesized, each PDI-4 subscale had higher correlations with the corresponding scale (eg, PDI-4 MDE with PHQ-9) than with any other scale. Correlations were positive and moderate to high among all scales (minimum of 0.33 between both PDI-4 GAD and PDI-4 MDE with the MDQ), which is consistent with the large amount of overlapping symptoms observed between the PHQ-9, HADS-A, CAARS-S, and MDQ (see Figure 1).

**Table 2:** Correlations between the four PDI-4 scales and corresponding diagnosis scales (PHQ-9, HADS-A, CAARS-S, and MDQ)

	**PDI-4 GAD**	**PDI-4 MDE**	**PDI-4 ADHD**	**PDI-4 Mania**
HADS-A	0.76	0.72	0.66	0.52
PHQ-9	0.75	0.87	0.68	0.46
CAARS-S	0.57	0.54	0.75	0.59
MDQ	0.33	0.33	0.46	0.63

Abbreviations: *ADHD* = attention deficit hyperactivity disorder; *CAARS-S* = Conners’ Adult ADHD Rating Scale Self Report; *HADS-A* = Hospital Anxiety and Depression Scale-Anxiety subscale; *MDE* = major depressive episode; *MDQ* = Mood Disorder Questionnaire; *PDI-4* = Provisional Diagnostic Instrument; *PHQ-9* = Patient Health Questionnaire for depression.

Note: Pearson correlation coefficients are reported. All correlations are significant, p<0.001.

The operating characteristics of the PDI-4 in this sample are provided in Table 3. Note that the gold standard in each case was compared to published cutoff points from the corresponding rating scale (HADS-A, PHQ-9, CAARS, and MDQ) and not to a diagnostic interview by a clinician, which was unavailable here. Sensitivities and positive predictive values were moderate to high (sensitivities ranging from 0.52 to 0.70; positive predictive values, from 0.44 to 0.72). Specificities and negative predictive values were high, ranging from 0.86 to 0.92 and 0.86 to 0.96, respectively, indicating the potential value of the PDI-4 to rule out diagnoses.

**Table 3:** Operating characteristics of PDI-4 based diagnosis relative to diagnosis based on corresponding rating scales

		**Operating Characteristics of PDI-4 Scale Using the Comparator Scale Diagnostic Cutoff**			
**PDI-4 Scale**	**Comparator Scale Diagnostic Cutoff**	**Sensitivity (95% CI)**	**Specificity (95% CI)**	**PPV (95% CI)**	**NPV (95% CI)**
PDI-4-GAD	HADS-A (≥ 14)	0.64 (0.54, 0.74)	0.92 (0.90, 0.93)	0.44 (0.35, 0.52)	0.96 (0.95, 0.97)
PDI-4-MDE	PHQ-9 (≥ 12)	0.69 (0.64, 0.75)	0.90 (0.88, 0.92)	0.72 (0.66, 0.77)	0.89 (0.87, 0.91)
PDI-4-ADHD	CAARS-S (≥ 28)	0.70 (0.62, 0.77)	0.87 (0.85, 0.89)	0.49 (0.42, 0.55)	0.94 (0.93, 0.96)
PDI-4-Mania	MDQ (≥ 7)	0.52 (0.46, 0.59)	0.86 (0.83, 0.88)	0.52 (0.46, 0.59)	0.86 (0.83, 0.88)

Abbreviations: *ADHD* = attention deficit hyperactivity disorder; *CAARS-S* = Conners’ Adult ADHD Rating Scale Self Report; *CI* = confidence interval; *HADS-A* = Hospital Anxiety and Depression Scale-Anxiety subscale; *GAD* = generalized anxiety disorder; *MDE* = major depressive episode; *MDQ* = Mood Disorder Questionnaire; *NPV* = negative predictive value; *PDI-4* = Provisional Diagnostic Instrument; *PHQ-9* = Patient Health Questionnaire for depression; *PPV* = positive predictive value.

**Figure 2:**
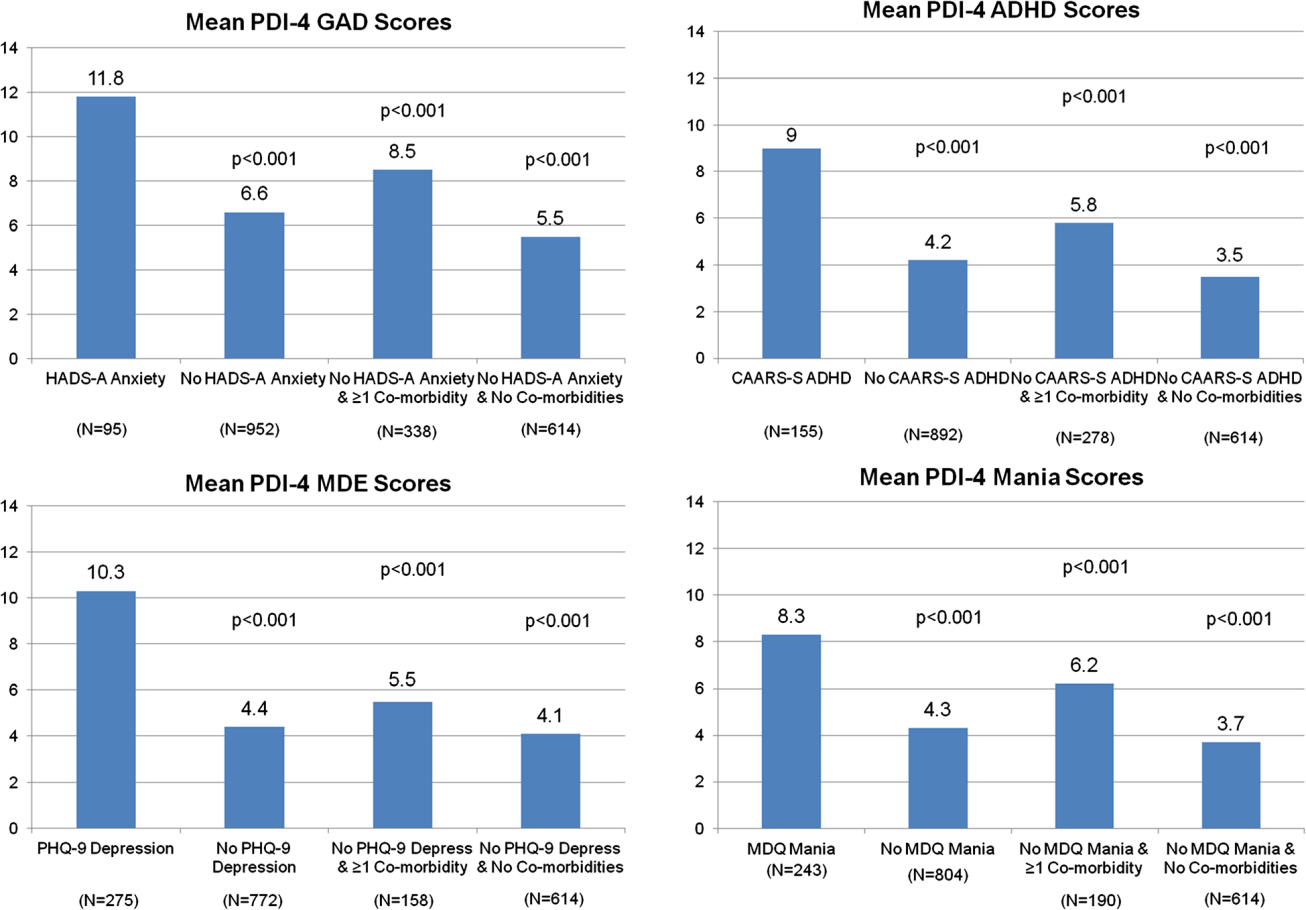
**Known groups validity: summary of mean PDI-4 subscale scores in cohorts diagnosed with other rating scales.** Abbreviations: ADHD = attention deficit hyperactivity disorder; CAARS-S = Conners’ Adult ADHD Rating Scale Self Report; Depress = depression; GAD = generalized anxiety disorder; HADS-A = Hospital Anxiety and Depression Scale-Anxiety subscale; MDE = major depressive episode; MDQ = Mood Disorder Questionnaire; PDI-4 = Provisional Diagnostic Instrument; PHQ-9 = Patient Health Questionnaire. P-values are from contrasts among the cohorts (comparing to the left most histogram bar) from the ANOVA model with the corresponding PDI-4 scale as the dependent variable and the following independent variables in the model: diagnostic cohorts, age, gender, and race. The Diagnostic Cohorts variable was a categorical grouping of patients into 3 categories: 1) patients meeting the corresponding cutoff score (PHQ-9 score ≥ 12 if PDI-4 MDE is the dependent variable, HADS-A score ≥ 14 if PDI-4 GAD is the dependent variable, CAARS-S score ≥ 28 if PDI-4 ADHD is the dependent variable, or MDQ score ≥ 7 with functioning item score of ≥ 3 if PDI-4 Mania is the dependent variable); 2) patients not meeting the cutoff score for the corresponding disease but who do meet at least 1 of the other 3 cutoff scores (denoted by “comorbidity” in the graphs); and 3) patients not meeting the cutoff score for the corresponding disease or any of the other 3 diagnoses.

Figure 2 summarizes the mean PDI-4 subscale scores based on cohorts used in the known groups (discriminant) validity analyses. As hypothesized, each subscale of the PDI-4 was able to discriminate between groups based on an independent assessment for each of the corresponding diagnoses (p<0.05 in all cases). For instance, PDI-4 GAD scores were statistically significantly higher in participants who met the HADS-A criteria for anxiety than in participants who did not (p<0.001). Mean subscale score differences between cohorts with and without the diagnosis were large in all cases, ranging from 3.3 (mania) to 5.5 (MDE). In addition, PDI-4 GAD scores were statistically significantly higher in participants who met the HADS-A criteria for anxiety than in 1) participants who did not meet the HADS-A criteria but did meet criteria for 1 of the other diagnoses (PHQ-9 for MDE, CAARS for ADHD, and MDQ for mania), and 2) participants who did not meet HADS-A criteria nor criteria for any of the other diagnoses. When comparing to patients with co-morbidities (eg, for anxiety, looking at patients with anxiety versus those without anxiety but with 1 of the other 3 diagnoses), cohort differences on the PDI-4 scales were smaller (range 2.0 to 4.8) but remained statistically significantly different in all analyses.

## Discussion

In this cross-sectional cross-validation study, concurrent validity and known groups validity of the PDI-4 were examined using an established closed-panel internet survey sample. Data on over 1,000 patients completing the PDI-4 and comparator scales were assessed.

The evidence supporting the concurrent validity of the scale was strong. Correlations with other validated scales measuring the same concepts were high, as expected. In fact, the observed correlations were extremely close to the correlations observed in the initial site-based study of the PDI-4. This suggests the scales perform similarly in this population and with this mode of administration as seen in the original PDI-4 office site-based paper approach. Although no confirmation of diagnoses using the gold standard of trained clinician interviews was available, relative to validated patient-rated scales, the observed specificity values were high, sensitivity values were moderate to high, and negative predicted values were high. Note that the positive predictive values in this type of sample – where patients are self-selected to have greater rates of mental health disorders – would be expected to be lower in a general population sample where the prevalence of mental disorders is lower [22].

The study also provided further support for the discriminant validity of the PDI-4. Large and statistically significant consistencies were observed between the PDI-4 in patients who did and did not meet diagnostic cutpoints for GAD, MDE, ADHD, and mania based on independent, validated scales for each diagnosis. Comparisons between groups of patients with a given validated scale-based diagnosis, for example, GAD based on HADS-A, and those without GAD but with a scale-based diagnosis of MDE, ADHD, or mania, highlighted the challenges clinicians face in making specific diagnoses with overlapping symptoms. Differences in PDI-4 summed subsection severity scores between such patient cohorts were much smaller, though the PDI-4 scores still demonstrated statistical separation between patients with and without a particular diagnosis in all cases.

These results are consistent with overlapping diagnostic symptom criteria for these 4 diagnoses as well as with partial overlap in pharmacologic treatments indicated for the treatment of the diagnoses. This underscores the need for careful clinician analysis which considers the possibility of multiple diagnoses in the selection of treatments since misdiagnosis and inappropriate treatment is potentially disastrous; for example, treatment of a major depressive episode in a bipolar I patient with an antidepressant alone could result in a manic episode. The PDI-4 is a tool designed to assist in diagnosis and to broaden diagnostic considerations, but it is not a substitute for clinical assessment.

One major limitation of this study was the lack of gold standard diagnostic interviews performed by trained clinicians. This meant that the operating characteristics (sensitivity, specificity) of the PDI-4 as well as the known groups validity analyses had to be assessed relative to other validated self-rating scales rather than the standard of using diagnostic interviews. It is unclear how the results may have differed had diagnostic interviews been performed and cohorts based on these data would have been available.

Another limitation of this study is the reliance on an internet sample of participants with self reported diagnoses. It is known that internet samples will differ to some unknown degree from a random sample of patients attending primary care centers. However, internet usage is increasing and thus “online samples” are becoming more like a general population sample in the US. Regardless, the PDI-4 validity evidence found in this specific study population may not generalize to the national primary care population.

## Conclusions

In total, these findings in a new sample and with a new mode of administration support prior office-based evidence that the PDI-4 appears to be a valid, brief self-rated instrument for assisting with the assessment of 4 common psychiatric diagnoses. It is important to note that given the large amount of symptom overlap in these diagnoses, such brief scales cannot replace thorough diagnostic evaluation by trained medical caregivers. However, the data suggest the PDI-4 can be a useful tool in the overall process of appropriate diagnosis of mental disorders.

## Competing interests

Authors DEF, JPH, and RWS were full-time employees and minor stockholders of Eli Lilly and Company when this research was conducted. Author EMS is a full-time employee of Harris Interactive.

## Authors’ contributions

DEF participated in the design, analysis, interpretation of results, and drafting of the manuscript. JPH participated in the design, interpretation, and drafting of the manuscript. EMS participated in the design, analysis, and interpretation of results. RWS conceived of the study, participated in the design, analysis, and interpretation of the results. All authors read and approved the final manuscript.

## Pre-publication history

The pre-publication history for this paper can be accessed here:

http://www.biomedcentral.com/1471-2296/13/104/prepub

## Supplementary Material

Additional file 1 Provisional Diagnostic Instrument (PDI-4). Click here for file
