# Supplementary material for: A cross-validation of the provisional diagnostic instrument (PDI-4)
Source: BMC Fam Pract. 2012 Oct 15;13:104. doi: 10.1186/1471-2296-13-104 (PMC3515462; doi:10.1186/1471-2296-13-104)
Supplement: Additional file 1 — Provisional Diagnostic Instrument (PDI-4). [file 1471-2296-13-104-S1.doc]

PDI-4 with scoring overlay

| **Provisional Diagnostic Instrument (PDI-4)** | | | | | | |
| --- | --- | --- | --- | --- | --- | --- |
| **Instructions: Answer each question below by placing an “X” under the ONE response that best describes you.** | | | | | | |
| **How often over the PAST 30 DAYS have you been**  **bothered by . . .** | **Never** | **Rarely** | **Some-times** | **Most of the Time** | **All of the Time** |  |
| Having trouble controlling your worry? |  |  |  |  |  | **GAD** |
| Becoming more fatigued than usual? |  |  |  |  |  |
| Having trouble concentrating or with your mind going blank? |  |  |  |  |  |
| Worrying much more than needed about everyday activities? |  |  |  |  |  |
| **How often over the PAST 2 WEEKS have you been**  **bothered by . . .** | **Never** | **Rarely** | **Some-times** | **Most of the Time** | **All of the Time** |  |
| Having less interest and enjoyment than usual? |  |  |  |  |  | **MDE** |
| Feeling so depressed nothing could cheer you up? |  |  |  |  |  |
| Feeling worthless or guilty? |  |  |  |  |  |
| Having trouble sleeping or with sleeping too much? |  |  |  |  |  |
| **How often over the PAST 6 MONTHS have you been**  **bothered by . . .** | **Never** | **Rarely** | **Some-times** | **Often** | **Very often** |  |
| Avoided or delayed getting started when a project required a lot of thought? |  |  |  |  |  | **ADHD** |
| Felt overly active and compelled to do things, like you were driven by a motor? |  |  |  |  |  |
| Made careless mistakes on boring/difficult tasks? |  |  |  |  |  |
| Left your seat in meetings in which you should remain seated? |  |  |  |  |  |
| **How often DURING YOUR LIFETIME have there been periods of time FOR AT LEAST A WEEK when . . .** | **Never** | **Rarely** | **Some-times** | **Often** | **Very often** |  |
| You talked almost constantly? |  |  |  |  |  | **Mania** |
| You were extremely active and productive? |  |  |  |  |  |
| You felt so high or irritable you might lose control? |  |  |  |  |  |
| You had too much energy to be able to concentrate? |  |  |  |  |  |
| Have the symptoms in ***any*** of the sections above interfered with your usual daily functioning? |  |  |  |  |  |  |
